# Microangiopathy in multiple myeloma: a case of carfilzomib-induced secondary thrombotic microangiopathy successfully treated with plasma exchange and complement inhibition

**DOI:** 10.1186/s12882-023-03228-9

**Published:** 2023-06-19

**Authors:** Lorenzo Catanese, Katharina Link, Harald Rupprecht

**Affiliations:** 1 Department of Medicine V (Nephrology, Hypertensiology, Angiology and Rheumatology), Klinikum Bayreuth GmbH, Medizincampus Oberfranken, Preuschwitzer Str. 101, 95445 Bayreuth, Germany; 2 Friedrich-Alexander-University Erlangen-Nürnberg, Schloßplatz 4, 91054 Erlangen, Germany; 3 Kuratorium for Dialysis Bayreuth, Stolzingstraße 40, 95445 Bayreuth, Germany

**Keywords:** Case report, Thrombotic microangiopathy (TMA), Multiple myeloma (MM), Carfilzomib, Eculizumab

## Abstract

**Background:**

Thrombotic microangiopathy (TMA) is a potentially organ and life-threatening condition affecting patients with multiple myeloma (MM). Cases of proteasome inhibitor-induced TMA and specifically carfilzomib-induced TMA have been rarely reported and standards for diagnostic workup and treatment are not available.

**Case presentation:**

We describe a case of a male MM patient under salvage therapy including proteasome inhibitor carfilzomib following chemotherapy and autologous stem cell transplantation. The patient then developed acute kidney injury with clinical and laboratory signs of TMA. Hemodialysis became necessary and treatment with plasma exchange was initiated followed by therapy with C5 complement inhibitor eculizumab which led to amelioration of kidney function and hemolysis parameters.

**Conclusion:**

We report a patient with suspected proteasome inhibitor-induced secondary thrombotic microangiopathy that has been successfully treated with plasma exchange and eculizumab, a monoclonal antibody targeting complement factor C5.

## Background

Multiple myeloma (MM) is the second most common hematological malignancy. As new therapies are constantly being developed and 5-year-survival-rate is constantly rising, new diagnostic and therapeutic challenges regarding MM and its treatment arise [1]. Thrombotic microangiopathy (TMA) encompasses several distinct syndromes with different etiologies, all characterized by thrombocytopenia, microangiopathic hemolytic anemia (MHA), different degrees of acute kidney damage (AKI) and a variety of unspecific clinical symptoms [2]. Due to overlapping symptoms and clinical/laboratory findings, detection of TMA in MM can be challenging. TMA can be subdivided in three syndromes:

Thrombotic thrombocytopenic purpura (TTP) is caused by a reduced activity of the matrix metalloproteinase ADAMTS-13 (a disintegrin and metalloproteinase with a thrombospondin type 1 motif, member 13) due to congenital deficiency or acquired, inhibiting antibodies. ADAMTS-13 cleaves von-Willebrand-factor (vWF) multimers which have a key role in thrombocyte-endothelial interaction and thrombogenesis thus reducing thrombotic activity [3]. Absolute or functional deficiency of ADAMTS-13 leads to uncontrolled endothelial platelet adhesion and microvascular thrombosis. Untreated TTP has mortality rates up to 90% and immediate treatment with plasma exchange (PE) in order to remove circulating antibodies and replace with functional ADAMTS-13 and vWF, as well as additional treatment with caplacizumab and rituximab is crucial [4].

Hemolytic uremic syndrome (HUS) is a multicausal group of syndromes where a more pronounced acute kidney failure is a key feature in addition to thrombocytopenia and MHA [5]. Classical HUS is caused by enteropathogens that produce Shiga-like toxins, typically E. coli and Shigella. Mostly ingested through contaminated food Shiga toxin producing E. coli (STEC)-HUS can be diagnosed by stool analytics and polymerase chain reaction (PCR) analysis for mentioned pathogens and their toxins.

Atypical or complement mediated HUS (also defined by thrombocytopenia, MHA and typically AKI) may be caused by dysregulated alternative pathway of the complement system [6]. The most common cause is a pathogenic gene variant of a complement regulatory gene or less often autoantibodies targeting complement factors. TMA episodes are often triggered by certain events, e.g., pregnancy, drugs, malignancies, organ transplant or infection and can occur at any age.

Eculizumab, a humanized monoclonal antibody targeting complement factor C5, has been successfully used for many years in the treatment of complement mediated HUS [7]. C5 is a key component of the membrane attack complex.

TMA in multiple myeloma patients is broadly reported and must be etiologically divided as subcategories that have different outcomes and require specific therapy [8].

Proteasome inhibitor (PI)-induced TMA, transplant-associated TMA and TMA caused by multiple myeloma itself (first diagnosis or disease progression) are the commonly described disease entities. They can be subdivided in carfilzomib-induced aHUS, bortezomib-induced aHUS, bortezomib-induced TTP, transplant-associated TMA, MM-induced aHUS and MM-induced TTP [8].

## Case presentation

A male patient in his late 50s was admitted to the emergency room with fatigue and resting dyspnea. He also reported episodes of minor nasal and gingival bleedings that had occurred within the last two weeks.

The patient had been diagnosed with smoldering multiple myeloma type IgA-Kappa 5 years and 6 months ago. Within 20 months after diagnosis bone marrow infiltration had increased from 40 to 75%. Cytogenetic analysis had revealed 17p deletion. Therefore, induction therapy with bortezomib, cyclophosphamide and dexamethasone had been started. After two cycles cyclophosphamide had been discontinued due to adverse effects. Therapy was changed to bortezomib, doxorubicin, dexamethasone and lenalidomide resulting in very good partial remission. Two years and six months after the primary diagnosis autologous stem cell transplantation was performed and maintenance therapy with lenalidomide was initiated. After two years progressive disease was diagnosed and salvage therapy with isatuximab, carfilzomib and dexamethasone was started.

Addressing other preexisting conditions, he had received a partial lumbar nephrectomy due to clear cell renal cell carcinoma (tumor classification: tumor under 4 cm T1a; without evidence for any metastases N0 and M0; highly differentiated G1) approximately 4 years prior. Furthermore, he suffered from arterial hypertension and obesity.

### Investigations

Vital parameters were heart rate 82/min, oxygen saturation 99%; respiratory rate 30/min, arterial blood pressure 182/89 mmHg and auricular temperature 36,9 °C.

Urine sediment analysis showed five erythrocytes per high power field (HPF), numerous granulated casts and tubular epithelia. As urinary dip test for hemoglobin was highly positive and only few erythrocytes were present in sediment analysis, we hypothesized this being due to hemoglobinuria. Furthermore, the urine sediment was suggestive of acute tubular necrosis. Urine protein analysis, however, showed nephrotic range glomerular proteinuria (Table 1).

**Table 1:**
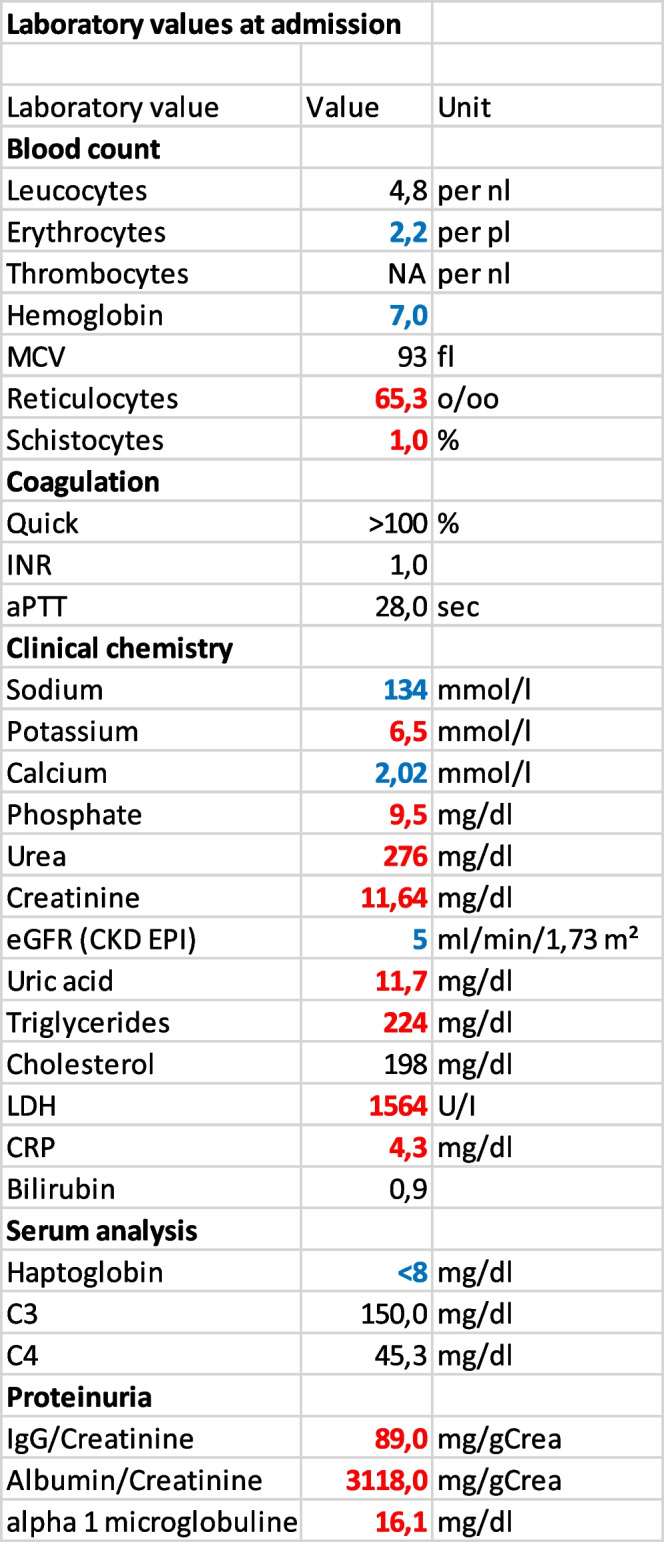
Laboratory values of the patient at hospital admission. Blue color of values indicates the value being under and red value above normal reference range

Blood gas analysis (Table 2) revealed metabolic acidosis and hyperkalemia. Serum creatinine was 11,64 mg/dl (Table 1) in contrast to laboratory tests two weeks prior to hospitalization which showed creatinine levels of 0,96 mg/dl corresponding to normal estimated glomerular filtration rate (eGFR).

**Table 2:**
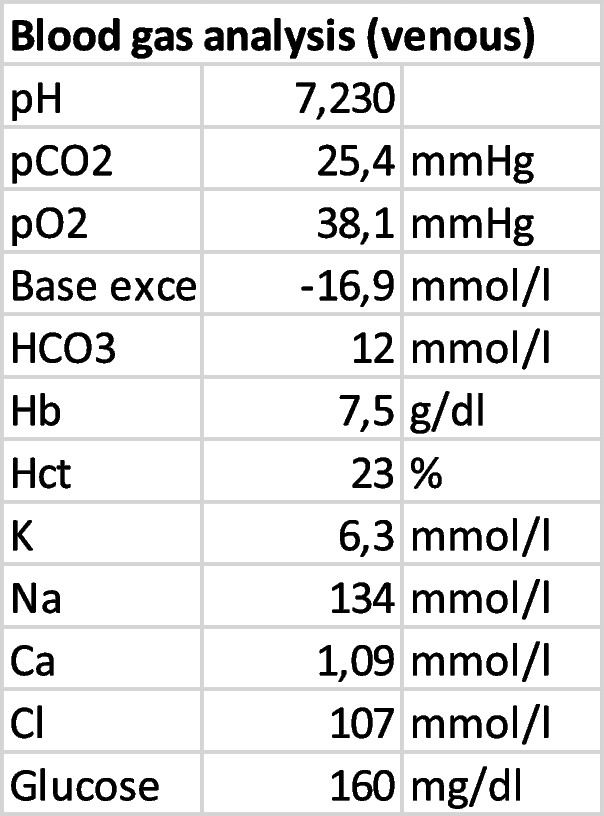
Venous blood gas analysis at admission

Elevated lactate dehydrogenase (LDH), decreased hemoglobin (decline from 13,9 to 7,0 g/dl within two weeks), undetectable haptoglobin and the presence of schistocytes in the performed blood smear were highly indicative of intravascular anemia. Thrombocytes were not measurable initially indicating massive thrombocytopenia (Table 1). Indirect Coombs test showed positive results without antibody specificity probably due to recent treatment with isatuximab. After treatment of patient blood with dithiothretiol, an inhibitor of isatuximab erythrocyte binding, Coombs testing was negative indicating non-immunohemolytic hemolysis. These findings combined with severe acute kidney injury, markedly elevated blood pressure and unspecific fatigue raised strong suspicion for the presence of thrombotic microangiopathy.

PLASMIC score, which incorporates platelet count, serum creatinine, the presence of hemolysis, history of cancer or organ transplantation, mean corpuscular volume, and international normalized ratio (INR), for thrombotic thrombocytopenic purpura was 4 indicating low risk for TTP. ADAMTS-13 analysis was promptly initiated and available after one week. Antibodies against ADAMTS-13 were not detected and ADAMTS-13 activity was within normal range in enzyme-linked immunoassay (ELISA) thus excluding TTP as a differential diagnosis. Complement analysis showed no abnormal values (Table 3).

**Table 3:**
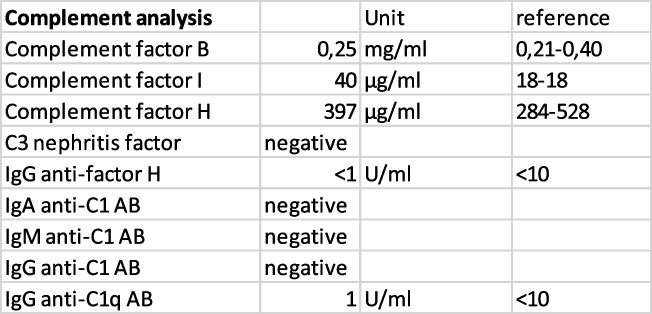
Complement analysis

A gastrointestinal PCR-Panel did not reveal potential Shiga toxin producing enteropathogenetic DNA.

### Differential diagnosis

Exhibiting clear signs of thrombocytopenia and microangiopathic hemolytic anemia, the two hallmarks of thrombotic microangiopathy, imminent evaluation of differential diagnosis is crucial as treatments differ and immediate initiation of treatment is essential to enhance outcomes and prevent irreversible organ damage or death.

Thrombotic thrombocytopenic purpura is characterized by decreased ADAMTS-13 activity or autoantibodies targeting ADAMTS-13. Laboratory workup regarding its activity is usually available after several days and cannot be used for quick assessment. TTP probability has to be established rapidly as mortality in untreated cases can be up to 90%. Bendapudi et al. have published a scoring system called PLASMIC-Score containing seven readily available items derived from patient history or routine laboratory workup for rapid assessment of TTP probability [9]. High scores are associated with high probability for TTP (Score 6–7 indicates > 72% probability of TPP) and the authors recommend plasma exchange upon reaching 6 points and the treatment with caplacizumab, a bivalent nanobody binding the A1 domain of vWF and inhibiting its interaction with thrombocytes thus preventing intravascular, vWF-mediated thrombocyte adhesion.

Distinguishing typical (Shiga toxin-mediated) and atypical HUS is also relevant as typical HUS is usually treated with supportive care while aHUS requires complement inhibition in order to prevent irreversible organ damage and decrease risk of mortality.

Typical HUS is caused by enterotoxic effects of Shiga-toxin producing enteropathogens like Shigella or Shiga toxin producing E. coli (STEC). This can be assessed either by direct analysis of serological evidence of these toxins or by detection of DNA fragments of enteropathogens that usually produce these toxins by PCR analysis.

There are no secure diagnostical parameters that are pathognomonic for aHUS. Though complement analysis (C3, C4 and CH50) can help diagnosis in certain cases, there is plenty of evidence of aHUS (confirmed by genetic analysis) without abnormalities in complement levels. Therefore, the clinically accepted approach to diagnosing aHUS when strong clinical suspicion for TMA exists, is to exclude TTP and STEC-HUS while empirically using plasma exchange and eculizumab under given conditions.

In this particular case it was important to distinguish drug-induced TMA from TMA associated with multiple myeloma which is an important differential diagnosis. This differentiation can be challenging in certain cases, and it cannot always be ruled out with complete certainty. However, in our case there a several factors indicating carfilzomib as the causative agent for TMA: the temporal association of carfilzomib treatment and TMA as well as the improvement during discontinuation of carfilzomib, the fact that the patient was not in a progressive state of multiple myeloma and light chain load was low due to ongoing treatment and the fact that so far, no recurrence of TMA was observed after discontinuation of carfilzomib.

### Treatment

Due to hyperkalemia, metabolic acidosis, and resting dyspnea with clinical signs of hypervolemia the patient was immediately treated with daily hemodialysis. After exclusion of TTP and typical HUS we considered the patient to have secondary TMA after treatment with carfilzomib. Therapy with daily plasma exchange sessions (30 ml/kg bodyweight) was initiated.

Additionally, we started empirical therapy with 900 mg of eculizumab weekly for four weeks. Prophylactic antibiosis was started, and meningococcal vaccination was initiated.

Hemodialysis could be discontinued after six sessions within nine days of treatment.

Carfilzomib therapy was discontinued, and we strongly advised against further application of PIs.

### Outcome and follow-up

**Fig. 1:**
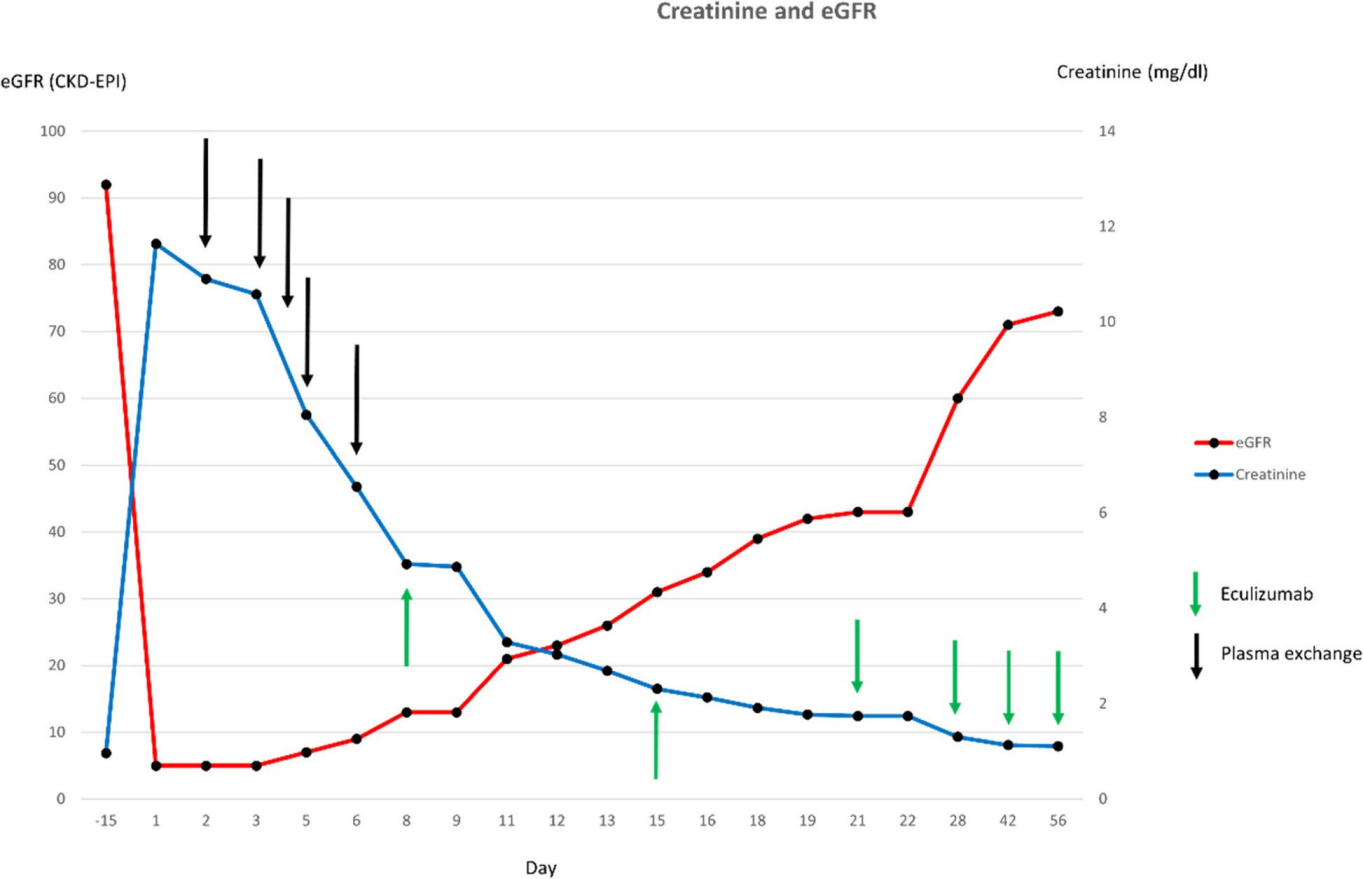
Creatinine(blue) and eGFR (CKD-EPI; ml/min/1.73m2) course of the patient over the duration of 56 days after diagnosis. Treatment with plasmapheresis is indicated with black arrows and treatment with eculizumab (first four doses of 900 mg and following doses of 1200 mg) is indicated with green arrows

Plasmapheresis and hemodialysis were discontinued after five and six sessions, respectively. Initially, eculizumab was given four times with a cumulative dose of 3600 mg followed by application of 1200 mg every two weeks for a total duration of 3 months. Hemolysis parameters improved and kidney function was steadily increasing. Proteinuria and hemoglobinuria were not detectable after four weeks of treatment. A graphic overview showing amelioration of kidney function throughout the treatment is given in Fig. 1. The patient is alive and well to the current moment (9 months after TMA). Kidney function was stable and hemolysis parameters inapparent after discontinuation of eculizumab treatment. The patient recently received allogenic stem cell transplantation.

## Discussion

In this case report, we describe a patient with a history of MM treated with chemotherapy and autologous stem cell transplantation currently under salvage therapy with carfilzomib, isatuximab and dexamethasone and planned allogenic stem cell transplantation. After the third cycle of chemotherapy the patient developed acute kidney injury, microangiopathic hemolytic anemia and thrombocytopenia which we diagnosed as carfilzomib-induced TMA. We successfully treated the patient with plasma exchange followed by empiric therapy with C5 antibody eculizumab. As cases like this one with severe kidney failure and MHA as well as with good response to plasma exchange and complement inhibition are rare, we believe that this case can help clinicians with diagnosis and therapeutic decisions. Furthermore, we give a short overview of TMA in the context of MM which has been a topic of recent scientific discussion and can be a diagnostical and therapeutical challenge with great impact on clinical outcomes.

As outlined above, differential diagnosis of TMA consists of (typical) Shiga toxin mediated hemolytic uremic syndrome, complement mediated TMA (atypical HUS), thrombotic thrombocytopenic purpura, coagulation mediated TMA and others [10]. The described case as drug induced TMA falls under the category of secondary TMA. There is a multitude of agents and conditions being described as causative of secondary TMA such as infections (not Shiga toxin), vaccination, tumors, malignant hypertension, autoimmune disease, metabolic disorders, drugs (e.g. quinin, chemotherapeutics, heroin, cocaine etc.) and others [11].

Thrombotic microangiopathy is an entity associated with multiple myeloma and monoclonal gammopathy. A comprehensive review about different forms of TMA associated with MM has been published recently [8]. Different forms of TMA have been associated with disease progression (direct effects of increasing M-protein), stem cell transplantation and drugs used in treatment of multiple myeloma. Exact mechanisms of the individual causes of TMA have yet to be understood. Notably, TMA has also been provisionally implemented as an associated lesion within the renal biopsy in monoclonal gammopathy of renal significance by the International Kidney and Monoclonal Gammopathy Research group and can be observed as first manifestation of the disease [12]. In the past, only case reports connecting TMA and MM were available. Ravindran et al. were the first to describe the association in a retrospective study of 146 patients with clinical diagnosis of TMA. They found monoclonal gammopathy in 13,7% of TMA patients and 21% of patients older than 50 years. The prevalence in these older TMA patients was fivefold higher than the expected prevalence in that age group [13]. Different potential mechanisms how the monoclonal immunoglobulin can induce TMA are discussed in the paper like direct endothelial damage caused by the Ig, interference with fibrin structure or interference with thromboregulatory proteins.

Determining the cause and form of TMA in MM patients proves to be challenging but is essential for therapeutic decisions. Decision for early plasma exchange is often taken because of its benefits for several TMA subforms but foremost because definite exclusion of TTP is not immediately possible in clinical practice due to the delay caused by assessment of ADAMTS-13 activity and antibodies. In our case five sessions of plasma exchange were performed already resulting in recovery of kidney function and amelioration of hemolysis parameters.

Carfilzomib-induced aHUS is a very rare complication in multiple myeloma patients with very few case reports available [14–16]. Eculizumab is a well-established drug used in treatment of aHUS and paroxysmal nocturnal hematuria. It directly interferes with the alternative complement pathway. Case reports of successful treatment of drug induced TMA with eculizumab exist with different causative agents such as Tacrolimus, Gemcitabine and also carfilzomib [17–23]. This underlines the role of potential complement involvement in the etiology of drug induced TMA.

Complement mutations which are often present in aHUS (50–60%) are also present in TMA of other causes such as TMA caused by infections, pregnancy, or malignant hypertension. Interestingly, complement mutations in cases of drug induced TMA were found in less than one percent of patients [24]. Therefore, we did not perform genetic testing of complement mutations in our patient.

### Limitations

Due to severe thrombocytopenia kidney biopsy could not be performed. Thus, histopathological correlates of thrombotic microangiopathy within the renal vasculature could not be determined. The fact that urine sediment analysis showed signs of acute tubular necrosis and urine protein analysis showed nephrotic range glomerular proteinuria potential overlapping mechanisms of acute kidney injury could not be ruled out completely. Strong clinical and laboratory findings indicating thrombotic microangiopathy and exclusion of TTP and STEC-HUS strongly indicate that the diagnosis of drug-induced TMA was correct.

Furthermore, to this point genetic analysis regarding mutations in complement genes were not performed. So, we cannot rule out that the patient had pathogenic complement variants and carfilzomib was a necessary second hit to trigger severe complement activation.

## Conclusion

In this case report we present a MM patient diagnosed with carfilzomib-induced TMA successfully treated with plasma exchange and eculizumab. Fast assessment of TMA cause especially in MM patients is crucial for therapeutic decision and prevention of irreversible organ damage and death. Early empiric eculizumab therapy proves to be a feasible treatment to increase renal outcomes.

### Learning points

Carfilzomib-induced TMA should be considered as a differential diagnosis in MM patients with acute kidney injury and MHA. Immediate treatment with plasma exchange and complement inhibitors like eculizumab can prevent organ damage and death and should be considered as a feasible treatment option. Pathophysiological mechanisms of TMA in the context of MM and especially of carfilzomib-induced TMA are not well understood and the involvement of complement dysregulation require further investigation.

## Data Availability

Not applicable.

## Abbreviations

ADAMTS-13: A disintegrin and metalloproteinase with a thrombospondin type 1 motif, member 13
aHUS: Atypical hemolytic uremic syndrome
AKI: Acute kidney injury
aPTT: Activated partial thromboplastin clotting time.
C5: Complement component 5
Ca: Calcium
CKD-EPI: Chronic Kidney Disease Epidemiology Collaboration
Cl: Chloride
CRP: C-reactive protein
eGFR: Estimated glomerular filtration rate
ELISA: Enzyme-linked immunoassay
Hb: Hemoglobin
Hct: Hematocrit
HPF: High power field
HUS: Hemolytic uremic syndrome
Ig: Immunoglobulin
INR: International normalised ratio
K: Potassium
LDH: Lactate dehydrogenase
MCV: Mean corpuscular volume
MHA: Microangiopathic hemolytic anemia
MM: Multiple myeloma
Na: Sodium
PCR: Polymerase chain reaction
PE: Plasma exchange
PLASMIC: Platelet count; combined hemoLysis variable; absence of Active cancer; absence of Stem-cell or solid-organ transplant; MCV; INR; Creatinine
STEC-HUS: Shiga toxin-producing Escherichia coli – hemolytic uremic syndrome
TMA: Thrombotic microangiopathy
TTP: Thrombotic thrombocytopenic purpura
vWF: Von Willebrand factor

## References

[1] Moreau P, Attal M, Facon T (2015). Frontline therapy of multiple myeloma. Blood.

[2] Go RS, Winters JL, Leung N, Murray DL, Willrich MA, Abraham RS (2016). Thrombotic Microangiopathy Care Pathway: A Consensus Statement for the Mayo Clinic Complement Alternative Pathway-Thrombotic Microangiopathy (CAP-TMA) Disease-Oriented Group. Mayo Clin Proc.

[3] Sukumar S, Lämmle B, Cataland SR (2021). Thrombotic Thrombocytopenic Purpura: Pathophysiology, Diagnosis, and Management. J Clin Med.

[4] Dane K, Chaturvedi S (2018). Beyond plasma exchange: novel therapies for thrombotic thrombocytopenic purpura. Hematol Am Soc Hematol Educ Program.

[5] Karpman D, Loos S, Tati R, Arvidsson I (2017). Haemolytic uraemic syndrome. J Intern Med.

[6] Raina R, Krishnappa V, Blaha T, Kann T, Hein W, Burke L (2019). Atypical Hemolytic-Uremic Syndrome: An Update on Pathophysiology, Diagnosis, and Treatment: Update on Atypical Hemolytic Uremic Syndrome. Ther Apher Dial.

[7] Mache CJ, Acham-Roschitz B, Frémeaux-Bacchi V, Kirschfink M, Zipfel PF, Roedl S (2009). Complement inhibitor eculizumab in atypical hemolytic uremic syndrome. Clin J Am Soc Nephrol.

[8] Portuguese AJ, Gleber C, Passero FC, Lipe B (2019). A review of thrombotic microangiopathies in multiple myeloma. Leuk Res.

[9] Bendapudi PK, Hurwitz S, Fry A, Marques MB, Waldo SW, Li A (2017). Derivation and external validation of the PLASMIC score for rapid assessment of adults with thrombotic microangiopathies: a cohort study. Lancet Haematol.

[10] Barbour T, Johnson S, Cohney S, Hughes P (2012). Thrombotic microangiopathy and associated renal disorders. Nephrol Dial Transplant.

[11] Nester CM, Barbour T, de Cordoba SR, Dragon-Durey MA, Fremeaux-Bacchi V, Goodship THJ (2015). Atypical aHUS: State of the art. Mol Immunol.

[12] Leung N, Bridoux F, Batuman V, Chaidos A, Cockwell P, D’Agati VD (2019). The evaluation of monoclonal gammopathy of renal significance: a consensus report of the International Kidney and Monoclonal Gammopathy Research Group. Nat Rev Nephrol.

[13] Ravindran A, Go RS, Fervenza FC, Sethi S (2017). Thrombotic microangiopathy associated with monoclonal gammopathy. Kidney Int.

[14] Haddadin M, Al-Sadawi M, Madanat S, Tam E, Taiwo E, Luhrs C (2019). Late Presentation of Carfilzomib Associated Thrombotic Microangiopathy. Am J Med Case Rep.

[15] Darwin A, Malpica L, Dhanoa J, Hashmi H (2021). Carfilzomib-induced atypical haemolytic uraemic syndrome: a diagnostic challenge and therapeutic success. BMJ Case Rep.

[16] Rassner M, Baur R, Wäsch R, Schiffer M, Schneider J, Mackensen A (2021). Two cases of carfilzomib-induced thrombotic microangiopathy successfully treated with Eculizumab in multiple myeloma. BMC Nephrol.

[17] Gosain R, Gill A, Fuqua J, Volz LH, Kessans Knable MR, Bycroft R (2017). Gemcitabine and carfilzomib induced thrombotic microangiopathy: eculizumab as a life-saving treatment. Clin Case Rep.

[18] Portuguese AJ, Lipe B (2018). Carfilzomib-induced aHUS responds to early eculizumab and may be associated with heterozygous CFHR3-CFHR1 deletion. Blood Adv.

[19] Moliz C, Gutiérrez E, Cavero T, Redondo B, Praga M (2019). Eculizumab as a treatment for atypical hemolytic uremic syndrome secondary to carfilzomib. Nefrol Engl Ed.

[20] Casiez C, Pica GM (2020). Bally S [Carfilzomib-induced haemolytic and uremic syndrome: Favorable outcome with eculizumab]. Nephrol Ther.

[21] Bhutani D, Assal A, Mapara MY, Prinzing S, Lentzsch S (2020). Case Report: Carfilzomib-induced Thrombotic Microangiopathy With Complement Activation Treated Successfully With Eculizumab. Clin Lymphoma Myeloma Leuk.

[22] Gabr JB, Bilal H, Mirchia K, Perl A (2020). The Use of Eculizumab in Tacrolimus-Induced Thrombotic Microangiopathy. J Investig Med High Impact Case Rep.

[23] Grall M, Daviet F, Chiche NJ, Provot F, Presne C, Coindre J-P (2021). Eculizumab in gemcitabine-induced thrombotic microangiopathy: experience of the French thrombotic microangiopathies reference centre. BMC Nephrol.

[24] Palma LMP, Sridharan M, Sethi S (2021). Complement in Secondary Thrombotic Microangiopathy. Kidney Int Rep.

